# Identifying a Test to Monitor Weightlifting Performance in Competitive Male and Female Weightlifters

**DOI:** 10.3390/sports6020046

**Published:** 2018-05-23

**Authors:** S. Kyle Travis, Jacob R. Goodin, George K. Beckham, Caleb D. Bazyler

**Affiliations:** 1 Department of Sport, Exercise, Recreation, and Kinesiology, East Tennessee State University, Johnson City, TN 37614, USA; goodinj@etsu.edu (J.R.G.); bazyler@etsu.edu (C.D.B.); 2 Kinesiology Department, California State University, Seaside, CA 93955, USA; gbeckham@csumb.edu

**Keywords:** weightlifters, squat jump, countermovement jump, isometric mid-thigh pull, Sinclair, snatch, clean and jerk, jump height, rate of force development, peak force

## Abstract

Monitoring tests are commonly used to assess weightlifter’s preparedness for competition. Although various monitoring tests have been used, it is not clear which test is the strongest indicator of weightlifting performance. Therefore, the purpose of this study was to (1) determine the relationships between vertical jump, isometric mid-thigh pull (IMTP) and weightlifting performance; and (2) compare vertical jumps to IMTP as monitoring tests of weightlifting performance in a large cohort of male and female weightlifters. Methods: Fifty-two competitive weightlifters (31 males, 21 females) participated in squat and countermovement jump testing (SJ, CMJ), and IMTP testing performed on force plates. All laboratory testing data was correlated to a recent competition where the athletes had attempted to peak. Results: Squat jump height (SJH) was the strongest correlate for men and women with the Sinclair Total (*r =* 0.686, *p* ≤ 0.01; *r =* 0.487, *p ≤* 0.05, respectively) compared to countermovement jump height (*r =* 0.642, *p ≤* 0.01; *r =* 0.413, *p =* 0.063), IMTP peak force allometrically scaled to body mass (*r =* 0.542, *p ≤* 0.01; *r =* −0.044, *p =* 0.851) and rate of force development at 200 ms (*r =* 0.066, *p =* 0.723; *r =* 0.086, *p =* 0.711), respectively. Further, SJH was a stronger correlate of relative weightlifting performance compared to IMTP peak force in females (*p =* 0.042), but not male weightlifters (*p =* 0.191). Conclusions: Although CMJ and IMTP are still considered strong indicators of weightlifting performance, SJH appears to be the most indicative measure of weightlifting performance across a wide-range of performance levels. Thus, SJH can be used as a reliable measure to monitor weightlifting performance in male and female weightlifters.

## 1. Introduction

In weightlifting, as in any sport, monitoring and assessing an athlete’s ability to recover and adapt is vital to ensure the athlete is prepared for competition [1]. Weightlifting performance depends heavily upon an athlete’s leg and hip strength, which are important for generating large ground reaction forces in a short time frame [2]. While 1-repetition maximum tests are often considered the gold-standard for assessing maximal strength, it is impractical for weightlifters to regularly perform a 1-repetition maximum snatch or clean and jerk in training. Instead, dynamic and isometric multi-joint performance tests have commonly been used to monitor and evaluate weightlifters [3,4,5,6,7,8,9,10,11,12,13,14,15].

Vertical jumps have been widely used to evaluate general athletic ability [16] and are biomechanically similar to weightlifting movements [8,9,10]. Strong relationships between squat jump and countermovement jump (SJ, CMJ) along with snatch and clean and jerk performance scaled to body mass (BM; *r =* 0.72 to 0.76) have been observed in national level male and female weightlifters [8]. Similar results have also been reported in international level weightlifters, with strong relationships between SJ (*r =* 0.66) and CMJ (*r =* 0.75) and the Sinclair Total (ST) [7]. However, some of these studies used contact mats, possibly leading to lower estimations of these relationships compared to force platforms or motion analysis software [7]. Therefore, an analysis with more robust instrumentation is needed to confirm these relationships [17,18].

The isometric mid-thigh pull (IMTP) is a viable monitoring test for weightlifters because both the maximal force and rate of force development (RFD) can be derived from the resultant force trace. Both of these variables are strongly related to weightlifting performance and are sensitive to changes in training volume-load [12,13,14,15]. Beckham et al. [15] found strong relationships between IMTP isometric peak force (IPF) and allometrically scaled IPF (IPFa) with snatch (*r =* 0.83, 0.62), clean and jerk (*r =* 0.84, 0.60), and competition total performance (*r =* 0.84, 0.79, respectively). The Sinclair Total and allometric scaling of competition results also showed a very strong relationship to IPF and IPFa, suggesting that maximum strength is an important factor even when BM is accounted for [12]. Similar relationships between IPF and snatch, clean and jerk, and absolute weightlifting performance (*r =* 0.93, 0.64, and 0.80, respectively) were also reported in a group of elite female weightlifters [13].

Although the weightlifters in the aforementioned studies were considered elite, the generalizability of the studies’ findings is limited due to small sample sizes (*n ≤* 12). Considering the potential value of vertical jumps and IMTP as monitoring tools for weightlifters, further research is needed to examine their relationship to weightlifting performance in a larger sample. To the authors’ knowledge, no studies to date have evaluated SJ, CMJ, and IMTP performance compared with absolute and scaled competition results in a large sample of weightlifters (*n* > 12). Therefore, the purpose of this study was to (1) determine the relationships between vertical jump, IMTP and weightlifting performance; and (2) compare vertical jumps to IMTP as monitoring tests of weightlifting performance in a large cohort of male and female weightlifters. We hypothesized that strong relationships would be observed between vertical jump, IMTP, and weightlifting performance in males and females. However, we also hypothesized that vertical jump performance would exhibit a stronger relationship than IMTP variables to weightlifting performance, and thus serve as a better monitoring test than IMTP.

## 2. Materials and Methods

This retrospective study sought to evaluate relationships between performance monitoring data and competition results from a weightlifting competition in which all lifters achieved a competition total. All subjects completed performance testing which ranged from 7.0 ± 5.2 days (range 2–18 days for males and 2–11 days for females) after a competition for which they peaked. Post-competition testing was completed as part of a long-term athlete monitoring program during a 2.5-week active recovery period to allow time for fatigue to dissipate. This study was granted a waiver of the requirement to obtain informed consent by the university’s institutional review board.

### 2.1. Athletes

A group of fifty-two weightlifters (31 males and 21 females) ranging from Level 1 to Master of Sport International Class participated in this study (Table 1). Within this group, there were national level (*n =* 20) and collegiate level weightlifters (*n =* 32), including weightlifters who compete at the International, Senior National, and Collegiate National levels. Each athlete’s weightlifting performance was classified according to Takano’s [19] classification system, from Master of Sport and below. According to each classification, the men and women were ranked accordingly: Master of Sport (*n =* 0 vs. *n =* 5, respectively), Candidate to Master of Sport (*n =* 9 vs. *n =* 6, respectively) and a range of Level 1, 2, and 3 individuals (*n =* 22 vs. *n =* 10, respectively) [19].

### 2.2. Hydration and Anthropometrics

Hydration status was evaluated prior to testing using a refractometer (ATOGO, Tokyo, Japan). Athletes were considered hydrated if urine specific gravity (USG) was <1.02. BM was measured with a calibrated digital scale to the nearest 0.1 kg (Tanita BF-350, Arlington Heights, IL, USA). Height measurements were assessed using a stadiometer (Cardinal Scale Manufacturing Co., Webb City, MO, USA) and recorded to the nearest 0.5 cm.

### 2.3. Standardized Warm-Up

Prior to testing, each athlete performed a standardized warm-up protocol of 25 jumping jacks, 1 set of 5 dynamic clean-grip mid-thigh pulls with 20 kg followed by 3 sets of 5 repetitions with 40 kg for females and 60 kg for males. Approximately 60 s of rest was allotted between sets.

### 2.4. Dynamic Vertical Jumps

All unloaded vertical jump testing was completed with a near weightless polyvinyl chloride (PVC) pipe (<1 kg). Squat jumps and CMJ were performed on dual force plates (Rice Lake Weighing Systems, Rice Lake, WI, USA; 1000 Hz sampling rate) on a custom built jumping platform covering an area of 91.4 × 91.4 cm. Athletes placed the PVC pipe on their shoulders similar to a back squat. For SJ, athletes were instructed to squat down to the ready position (90° knee angle measured using a handheld goniometer) and await the verbal command of “3, 2, 1, jump!” before jumping. Maximal effort jumps were preceded by warm-up jumps at 50% and 75% perceived effort. Athletes were given at least 60 s between SJ and CMJ tests. During the CMJ test, athletes were instructed to stand still and await the verbal command of “3, 2, 1, jump!” before performing a CMJ from a self-selected depth. A single warm-up CMJ at 75% perceived effort preceded maximal effort trials.

Jump height and peak power were selected as variables of interest because both exhibit strong relationships to weightlifting ability [7,8]. All vertical jump testing trials were recorded and analyzed using a custom analysis program (LabView 2010, National Instruments Co., Austin, TX, USA). Squat jump height (SJH) and CMJ height (CMJH) were estimated from flight time as previously described [18]. Peak power was determined as the maximal value obtained from the product of the force–time trace and derived velocity–time trace during the concentric phase of the jump. Peak power was allometrically scaled (PPa) to the lifter’s BM for both SJ (SJPPa) and CMJ (CMJPPa). The mean of two trials within a 2 cm difference in jump height was used for analysis. Additional trials were performed if the difference in jump height was greater than 2 cm. Test–retest reliability has been recently reported from our laboratory for JH (ICC = 0.93 to 0.99, CV = 2.08 to 7.32%) and PPa (ICC = 0.95 to 0.98, CV = 2.20% to 2.31%) [20,21].

### 2.5. Isometric Mid-Thigh Pull

The IMTP was performed on force plates (Rice Lake Weighing Systems, Rice Lake, WI, USA; 1000 Hz sampling rate) covering an area of 91.4 × 91.4 cm inside of a custom designed power rack in which a fixed bar could be adjusted for each subject’s appropriate mid-thigh position. Athletes were positioned into their respective power positions, similar to the start of the second pull of a clean with the knee angle set to 125 ± 5° measured with a handheld goniometer. Athletes were positioned with an upright torso and hip angle of approximately 145 ± 5° [1]. Athletes were then secured to the bar in their respective clean grip positions using lifting straps and athletic tape to remove grip strength as a limiting factor. A stable body position with a minimal pre-tension pull was initiated and verified on the force–time curve before receiving further verbal pull commands. Pre-tension was initiated by a verbal command—the tester stated “steady tension on the bar,” followed by a countdown “3, 2, 1, pull!” Athletes were instructed to continue to pull until the tester gave a downward hand signal. Each athlete completed warm-up attempts at 50% and 75% perceived effort before performing a maximal effort attempt. For the maximal effort pulls, athletes received loud, verbal encouragement to pull as “fast and hard” as possible. It has been suggested that giving verbal encouragement to achieve a higher IPF for each trial allows variables such as RFD to be measured appropriately [22]. If a countermovement on the force–time curve ≥200 N was observed before the pull or after a continuous pull, the athlete was given another attempt. The test was terminated if a consistent decrease or plateau in IPF was observed. A third trial was also administered if a difference of ≥250 N was observed between the first two trials. Athletes were given 2–3 min of rest between attempts.

Further procedures were in accordance with previous reports from our laboratory [1,15]. The start of each pull was identified by visual inspection [23]. Ground reaction forces were measured only in the vertical direction. IPF and average RFD from 0 to 200 ms (RFD200) were considered for the analysis. The monitoring of IPF and RFD is important in weightlifting given the need to produce high vertical ground reaction forces, particularly during the second pull phase of the clean [23]. IPF has been shown to have strong relationships with maximal strength, RFD200, and weightlifting performance [12,13,14,15]. All IPF values reported were gross values that were not offset by the athlete’s BM on the force plate. Thus, IPF was allometrically scaled to each athlete’s BM (IPFa) to determine relative IPF values using the equation: $\mathrm{IPF}\cdot {\mathrm{BM}}^{-\frac{2}{3}}$. Analog data from the force plate were amplified and conditioned (low-pass at 16 Hz) with a Transducer Techniques amplifier and conditioning module (Temecula, CA, USA). An analog-digital converter (DAQCard-6063E, National Instruments, Austin, TX, USA) was used for collection at 1000 Hz. A custom analysis program (LabView 2010, National Instruments Co., Austin, TX, USA) was used to analyze the mean of two trials at 100% effort within an IPF of 250 N apart. Interclass correlations (ICC) and coefficients of variation (CV) for IPF and RFD have been recently reported from our laboratory (ICC = 1.00, CV = 0.53%; ICC = 0.92, CV = 16.25%) [24].

### 2.6. Weightlifting Performance

Competition results for the snatch, clean and jerk, competition total, and ST were used to correlate absolute and scaled competition results to laboratory testing performance. The Sinclair formula is a polynomial equation used to adjust a weightlifter’s competition total to allow for comparison between lifters of different body mass; this formula is based on world records set during the previous Olympiad [25,26]. This method adjusts a lifter’s total to what it would be if they had a maximized body mass in the highest body weight category, given their current skill level. For the current Olympic cycle, the Sinclair formula is as follows:

$$\mathrm{Male}:\;\mathrm{ST}=\mathrm{Unadjusted}\;\mathrm{Total}\cdot 10^{\mathrm{AX}}{}^{2},\;\mathrm{where}\;X=\log_{10}\;\left(\mathrm{BM}\cdot 175.508^{-1}\right)\;\mathrm{and}\;A=0.751945030$$

$$\mathrm{Female}:\;\mathrm{ST}=\mathrm{Unadjusted}\;\mathrm{Total}\cdot 10^{\mathrm{AX}}{}^{2},\;\mathrm{where}\;X=\log_{10}\;\left(\mathrm{BM}\cdot 153.655^{-1}\right)\;\mathrm{and}\;A=0.783497476$$

If a lifter’s BM is greater than 175.508 kg (men) or 153.655 kg (women), then the absolute total should not be adjusted (i.e., the Sinclair total is the same as the unadjusted total).

### 2.7. Statistical Analysis

After the data set was scanned for outliers (cutoff: mean ± 3 SD), normality was assessed using the Shapiro–Wilks test. Pearson’s product moment zero-order correlations were calculated to determine the relationships between variables of the three testing methods (SJ, CMJ, IMTP) with absolute and scaled competition results. Fisher’s *r*-to-*z* transformation was used to compare correlation coefficients between each testing method and competition results while factoring in the shared variance between the testing variables [27]. A Holm’s sequential Bonferroni procedure was used to control Type I error inflation [28]. Correlation coefficients were evaluated using the following scale: 0.0–0.1 trivial, 0.1–0.3 weak, 0.3–0.5 moderate, 0.5–0.7 strong, 0.7–0.9 very strong, 0.9–1 nearly perfect [29]. The alpha criterion for all analyses was set at *p ≤* 0.05. SPSS software version 23 (IBM Co., Armonk, NY, USA) and Microsoft Excel (Microsoft Corporation, Redmond, WA, USA) were used to perform all statistical analyses.

## 3. Results

### 3.1. Male Weightlifters

A moderate relationship was found between IPF and the competition total (*r =* 0.495, *p* < 0.001), while strong relationships were found between IPFa and both the competition total (*r =* 0.571, *p* < 0.001) and ST (*r =* 0.542, *p* < 0.001). Squat jump height displayed strong relationships with both the competition total (*r =* 0.607, *p* < 0.001) and ST (*r =* 0.686, *p* < 0.001), and SJPPa showed moderate relationships with the competition total (*r =* 0.388, *p =* 0.031) and ST (*r =* 0.394, *p =* 0.028). Countermovement jump height displayed strong relationships with both the competition total (*r =* 0.541, *p* < 0.001) and ST (*r =* 0.642, *p* < 0.001). Within-sex comparisons for the male weightlifters showed the following relationships: ST–IPFa vs. ST–SJH (*z* = −0.873, *p =* 0.191), ST–IPFa vs. ST–CMJH (*z* = −0.578, *p =* 0.281), and ST–CMJH vs. ST–SJH (*z* = −0.295, *p =* 0.384). The observed statistical power ranged from 0.05 (RFD–ST) to 0.99 (SJH–Sinclair) for all male analyses.

### 3.2. Female Weightlifters

In female weightlifters, a moderate relationship was observed between SJH and ST (*r =* 0.487, *p =* 0.025), but this was not statistically significant after correcting for multiple comparisons (Table 2). Within-sex comparisons for the female weightlifters showed the following relationships: ST–IPFa vs. ST–SJH (*z* = −1.728, *p =* 0.042), ST–IPFa vs. ST–CMJH (*z* = −1.45, *p =* 0.074), and ST–CMJH vs. ST–SJH (*z* = −0.279, *p =* 0.39). The observed statistical power ranged from 0.04 (IPF-ST) to 0.69 (SJH-Sinclair) for all female analyses.

## 4. Discussion

**Figure 1:**
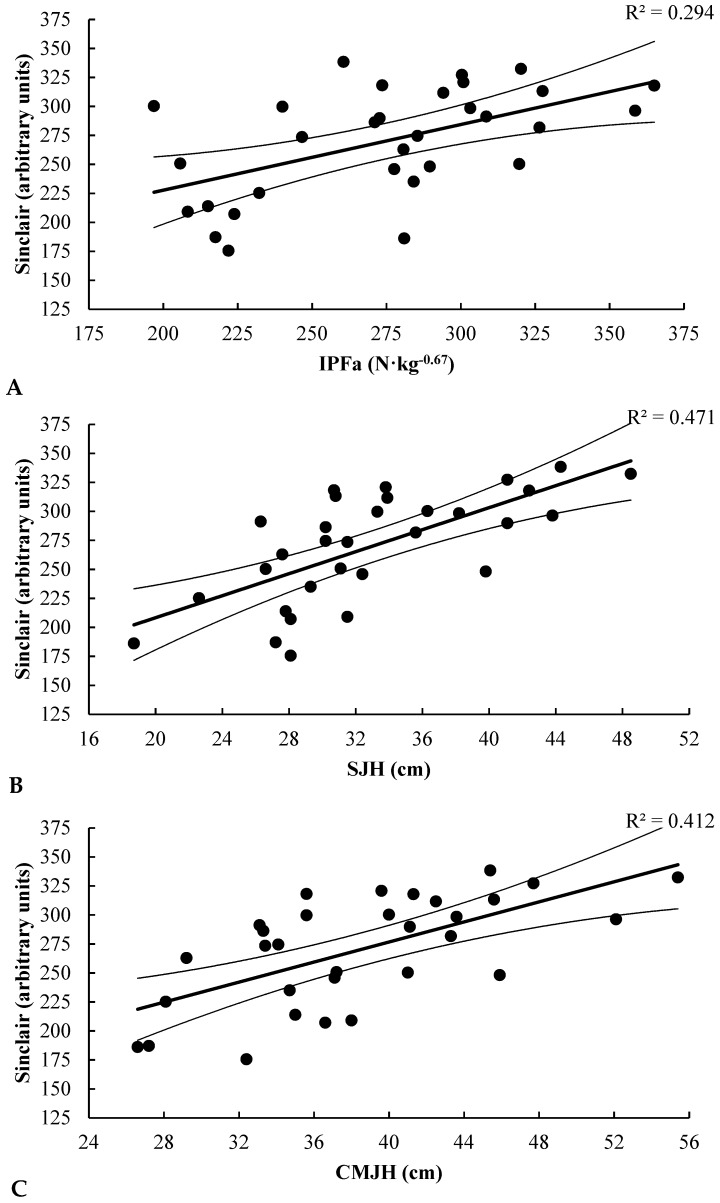
This figure shows the relationships between each testing variable and the Sinclair Total: (**A**) Relationship between IPFa and Sinclair Total for males; (**B**) Relationship between SJH and Sinclair Total for males; (**C**) Relationship between CMJH and Sinclair Total for males. IPFa: isometric peak force, allometrically scaled; SJH: squat jump height; CMJH: countermovement jump height.

**Figure 2:**
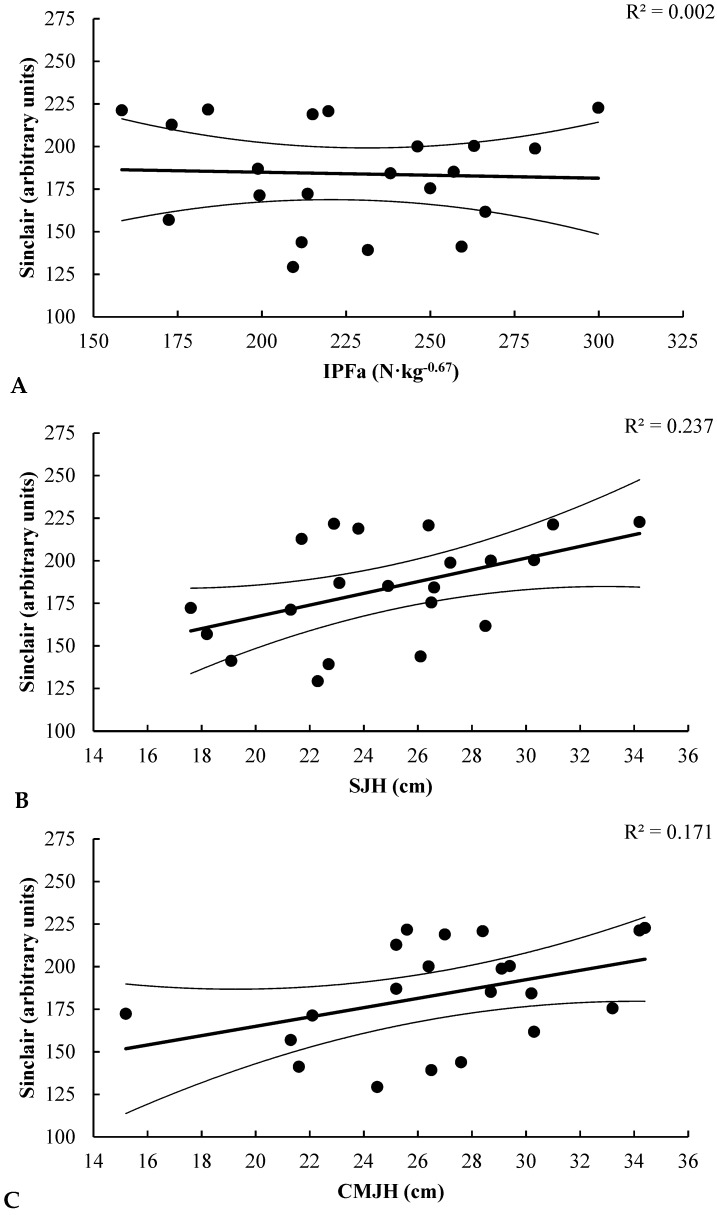
This figure shows the relationships between each testing variable and Sinclair Total: (**A**) Relationship between IPFa and Sinclair Total for females; (**B**) Relationship between SJH and Sinclair Total for females; (**C**) Relationship between CMJH and Sinclair Total for females. IPFa: isometric peak force, allometrically scaled; SJH: squat jump height; CMJH: countermovement jump height.

The purpose of the present study was to determine the relationships between vertical jump, IMTP, and weightlifting performance, and to compare vertical jumps with IMTP as monitoring tests of weightlifting performance in a large cohort of male and female weightlifters competing at various levels. The results of this study reject our original hypothesis that strong relationships would be observed between vertical jumps, IMTP, and weightlifting performance in male and female weightlifters. Only male weightlifters showed strong relationships between all performance tests and weightlifting performance (Figure 1). In contrast, females only showed a moderate relationship between jump height and weightlifting performance (Figure 2).

The results of the current analysis disagree with those of Beckham et al. [15] regarding the strength of the relationships between both IPF and IPFa to absolute (*r =* 0.838, *p ≤* 0.05; *r =* 0.610, *p ≤* 0.05) and scaled (*r =* 0.775, *p ≤* 0.05; *r =* 0.737, *p ≤* 0.05) weightlifting performances. However, the authors do agree that significant positive relationships exist. The differences between Beckham and colleagues’ findings and the current study may be attributed to sample size (10 males and 2 females vs. 31 males and 21 females, respectively), competition attempt selection, or athlete characteristics such as anthropometrics and weightlifting ability. However, the male athletes’ ST and IPFa were comparable between studies (258 ± 45, 288 ± 49 N·kg^−0.67^ vs. 270 ± 47, 274 ± 45 N·kg^−0.67^, respectively). Also, Beckham and colleagues’ analysis combined the results from the males and females together, whereas the current analyses separated them due to differences in the relationship between laboratory performance measures and weightlifting performance. The nonsignificant and trivial to weak relationships between competition performance and both IPF and IPFa in females may indicate that these athletes were limited by their weightlifting technique rather than strength influencing successfully completed lifts.

The current sample of female weightlifters is ranked below the female weightlifters used in similar studies that observed the same performance measurements [12,13,14]. In these studies [12,13,14], all of the female athletes (*n =* 7, *n =* 6, *n =* 6, respectively) were USA Weightlifting resident athletes who would be classified as Master of Sport International Class (i.e., the highest achievable classification). The female athletes used in these studies [12,13,14] were significantly stronger than the current sample with respect to average snatch (91 ± 7.7 kg), clean and jerk (112 ± 11.7 kg), and competition total (203 ± 18.7 kg). The group of female weightlifters in these studies outperformed the current sample on vertical jump SJH (29.0 ± 5.0 cm), CMJH (31.0 ± 4.0 cm), and RFD during IMTP (13,997.2 ± 1879.3 N·s^−1^). However, although comparable, this group underperformed on IMTP IPF (3510.0 ± 587.0 N) and IPFa (202.5 ± 35.5 N·kg^−0.67^). Thus, the current investigation agrees with Stone et al. [12] that correlations between measures of maximum strength and weightlifting performance are generally lower for women than men, yet weak and trivial correlations between IMTP and weightlifting performance for females were not expected. Therefore, women’s performance in weightlifting may be relatively more dependent on other factors such as mobility, technique, or speed under the bar rather than maximum strength (i.e., IPF, IPFa). With respect to the sample used in this investigation, although the females demonstrated greater strength capabilities on the IMTP compared to similar studies with more competitive female weightlifters, the average competition performance and ST was not sufficient to show stronger relationships. Therefore, the authors suggest that athletes with less competition experience, particularly female athletes, should primarily focus on weightlifting technique before shifting the training emphasis towards maximum strength. Stone et al. [12] state that weightlifting is not solely dependent on maximal strength, but is highly influenced by technical factors, possibly explaining the differences in relationships observed between previous findings and the current investigation.

In a recent review by Maffiuletti et al. [23] the authors stated that RFD seems to be strongly related to performances of sport-specific tasks such as ballistic actions for strength athletes, and is a sensitive concurrent indicator of neuromuscular function for accumulated fatigue. Further, isometric RFD, particularly at later time points (i.e., 200 and 250 ms), has been shown to be closely related to maximal isometric force and weightlifting performance [13,14,30,31]. In the current investigation, RFD showed trivial and weak relationships in males and females. One explanation for these findings may be that some or all of the athletes had not fully recovered during the active recovery period following the competition, and thus carried neuromuscular fatigue that impacted IMTP performance. There is evidence that suggests acute and chronic fatigue leads to a reduction in RFD, and peak force capabilities in elite female weightlifters [14] which may explain our findings. However, previous analyses from our laboratory have shown very strong correlations (*r* ≥ 0.94) between isometric data collected pre and post weightlifting competition as much as four months apart [15]. Nonetheless, the lack of strong or significant relationships between RFD and weightlifting performance is surprising and may point to unmeasured confounding factors within the current investigation.

Both SJ and CMJ performance correlated with weightlifting performance in males, with SJH displaying the strongest correlations to both competition total and ST. Squat jumps are unique in that they require both explosive strength ability and a rapid take-off velocity, and exhibit stronger correlations with maximal strength (e.g., 1-RM back squat, IMTP peak force) than CMJs [14,30,32]. Squat jump performance is also unique to weightlifters because it is considered to be biomechanically similar to the starting positions of the snatch and clean and jerk [10]. The act of holding the start position is thought to remove the contribution of series elastic components in the hip, knee, and ankle extensors so that the jumper must rely on concentric muscle action instead of the stretch-shortening cycle to generate high take-off velocities [33,34]. During what is known as “the double-knee bend”, weightlifters preload the leg extensor musculature during the transition into the second pull (i.e., the “power position”–the same position used in IMTP tests) [32]. Despite this potential invocation of the stretch-shortening cycle, a majority of the pull phase in both the snatch and clean involve concentric-only muscle actions. Even the double-knee bend, or shifting of the knees forward by extending the hips, may be a pause in knee extension rather than active knee flexion, resulting from a repositioning of the extensor muscles and spine to more optimally accelerate the barbell [35,36]. It may be that the strong correlations that SJH and SJPPa showed with the competition total and ST in males were due to these similarities in muscle action. The absence of these relationships in females may further highlight the sex-based differences in weightlifting technique in the current sample. An athlete with less experience and lower skill level may lack the ability to fully express their physical abilities in a competition setting.

Various physiological, biochemical, psychological, and neuromuscular measures have been used to monitor weightlifters [1]. While this study only focused on neuromuscular measures related to weightlifting performance, it is clear that athlete monitoring requires a broader ‘systems approach’. Training decisions should not be based on a single measurement. Nonetheless, a balance is needed to avoid over- or under-testing athletes. Thus, this study adds insight into weightlifting monitoring in a large sample of weightlifters across various skill levels. A few limitations, albeit difficult in practice, include the timing of testing relative to competition, the use of weightlifting competition results, and inclusion of only neuromuscular laboratory tests. However, these limitations are overcome by the large, heterogeneous sample of weightlifters, and the use of robust measurements of maximal strength and jumping performance.

## 5. Conclusions

Based on the results of the current analysis, SJH was the strongest correlate of weightlifting performance in the cohort’s most recent training and competition cycle. However, the relationship was weaker among female athletes which is likely due to discrepancies between strength levels and actual competition performance. Caution should be applied as longitudinal data is needed to confirm that SJ variables are sensitive to weightlifters’ training responses. Nonetheless, SJs can be used as a reliable measure to monitor weightlifting performance across a wide-range of weightlifting abilities.

## Figures and Tables

**Table 1:** Descriptive characteristics.

Characteristics	Male (*n =* 31)	Female (*n =* 21)
Age (years)	24.2 ± 4.7	21.0 ± 3.1
Height (cm)	174.0 ± 8.3	161.3 ± 7.4
BM (kg)	90.1 ± 14.6	66.5 ± 11.7
IPF (N)	5552.8 ± 986.6	3715.8 ± 613.7
IPFa (N·kg^−0.67^)	274.5 ± 44.9	226.1 ± 38.0
RFD200 (N·s^−1^)	10,820.4 ± 3838.5	6655.2 ± 2194.4
SJH (cm)	33.0 ± 6.8	24.9 ± 4.3
SJPPa (W·kg^−0.67^)	289.3 ± 91.6	185.0 ± 18.4
CMJH (cm)	38.4 ± 7.0	27.0 ± 4.6
CMJPPa (W·kg^−0.67^)	299.6 ± 98.6	191.6 ± 33.4
Snatch (kg)	99.9 ± 16.0	63.9 ± 11.4
Clean and Jerk (kg)	127.8 ± 21.2	80.5 ± 15.4
Total (kg)	227.4 ± 37.2	144.1 ± 26.9
Sinclair (AU)	269.9 ± 46.9	184.0 ± 30.4

All values are expressed as means ± SD. Notes: BM: body mass; IPF: isometric peak force; IPFa: isometric peak force, allometrically scaled; RFD200: rate of force development at 200 ms; SJH: squat jump height; SJPPa: squat jump peak power, allometrically scaled; CMJH: countermovement jump height; CMJPPa: countermovement jump peak power, allometrically scaled.

**Table 2:** Bivariate correlations.

Variable	Male (*n =* 31)		Female (*n =* 21)	
	Total	Sinclair	Total	Sinclair
IPF	0.495 *	0.256	0.247	−0.002
IPFa	0.571 *	0.542 *	−0.138	−0.044
RFD200	0.137	0.066	0.219	0.086
SJH	0.607 *	0.686 *	0.288	0.487
SJPPa	0.388	0.394	0.327	0.325
CMJH	0.541 *	0.642 *	0.217	0.413
CMJPPa	0.327	0.308	0.156	0.072

Note: Statistically significant at * *p* < 0.05. IPF: isometric peak force; IPFa: isometric peak force allometrically scaled; RFD200: rate of force development at 200 ms; SJH: squat jump height; SJPPa: squat jump peak power, allometrically scaled; CMJH: countermovement jump height; CMJPPa: countermovement jump peak power, allometrically scaled.
